# Effects of Climate Change on Land Cover Change and Vegetation Dynamics in Xinjiang, China

**DOI:** 10.3390/ijerph17134865

**Published:** 2020-07-06

**Authors:** Haochen Yu, Zhengfu Bian, Shouguo Mu, Junfang Yuan, Fu Chen

**Affiliations:** 1 School of Environment Science and Spatial Informatics, China University of Mining and Technology, Xuzhou 221116, China; haochen.yu@cumt.edu.cn (H.Y.); musg@cumt.edu.cn (S.M.); JunfangYuan@cumt.edu.cn (J.Y.); 2 School of Public Policy and Management, China University of Mining and Technology, Xuzhou 221116, China; 3 Low Carbon Energy Institute, China University of Mining and Technology, Xuzhou 221008, China

**Keywords:** NDVI, land-use/land-cover (LULC), climate change, climate extremes, Xinjiang

## Abstract

Since the Silk-road Economic belt initiatives were proposed, Xinjiang has provided a vital strategic link between China and Central Asia and even Eurasia. However, owing to the weak and vulnerable ecosystem in this arid region, even a slight climate change would probably disrupt vegetation dynamics and land cover change. Thus, there is an urgent need to determine the Normalized Difference Vegetation Index (NDVI) and Land-use/Land-cover (LULC) responses to climate change. Here, the extreme-point symmetric mode decomposition (ESMD) method and linear regression method (LRM) were applied to recognize the variation trends of the NDVI, temperature, and precipitation between the growing season and other seasons. Combining the transfer matrix of LULC, the Pearson correlation analysis was utilized to reveal the response of NDVI to climate change and climate extremes. The results showed that: (1) Extreme temperature showed greater variation than extreme precipitation. Both the ESMD and the LRM exhibited an increased volatility trend for the NDVI, with the significant improvement regions mainly located in the margin of basins. (2) Since climate change had a warming trend, the permanent snow has been reduced by 20,436 km^2^. The NDVI has a higher correlation to precipitation than temperature. Furthermore, the humid trend could provide more suitable conditions for vegetation growth, but the warm trend might prevent vegetation growth. Spatially, the response of the NDVI in North Xinjiang (NXC) was more sensitive to precipitation than that in South Xinjiang (SXC). Seasonally, the NDVI has a greater correlation to precipitation in spring and summer, but the opposite occurs in autumn. (3) The response of the NDVI to extreme precipitation was stronger than the response to extreme temperature. The reduction in diurnal temperature variation was beneficial to vegetation growth. Therefore, continuous concentrated precipitation and higher night-time-temperatures could enhance vegetation growth in Xinjiang. This study could enrich the understanding of the response of land cover change and vegetation dynamics to climate extremes and provide scientific support for eco-environment sustainable management in the arid regions.

## 1. Introduction

Arid and semi-arid regions encompass nearly 40% of the Earth’s land surface, where about 20% of the human population of the world live [1]. As one of the largest arid and semi-arid regions in the Northern hemisphere, Central Asia is particularly influenced by drought [2,3,4]. Vegetation dynamics could reflect the changes in the ecological environment to some extent [5,6,7], and it might be extremely sensitive to climate change in fragile eco-environment [8,9,10]. As a previous study has found, the contribution of climate change to grassland degradation could reach up to 47.9% in the arid and semi-arid regions of China [11]. Therefore, research on vegetation dynamics and its response to climate change remains a vital strategic task in arid and semi-arid regions [12,13,14,15,16].

The Normalized Difference Vegetation Index (NDVI) has been regarded as a reliable index to monitor vegetation dynamics [17,18,19]. Additionally, the NDVI product supported by the Moderate-resolution Imaging Spectroradiometer (MODIS) has been used to widely prove the effectiveness of vegetation data collection and analysis from regional to global scales [20,21,22]. Consequently, a considerable amount of research has been conducted to quantitatively analyze the responses of vegetation activity and climate change, and great progress has been achieved in this area [23]. Recent studies point out that precipitation is considered to be the vital limiting factor in vegetation growth in Central Asia [8,24]. Furthermore, some studies have also suggested that the temperature decrease in spring or autumn could limit vegetation growth [25,26,27]. However, the responses of different vegetation types to climate change show great disparity, which undoubtedly increases the difficulty of quantitative analysis [28,29,30].

Over the past few decades, some researchers have found a warmer and more humid pattern displayed in the arid regions of Central Asia [24,31], but some studies have come to the opposite conclusion [32,33]. Meanwhile, some studies have also proposed the presence of enhanced vegetation greenness in Central Asia [28], and further studies have documented a significant spatial heterogeneity [31,34] and seasonal diversity [25,35] in the vegetation greenness. Several studies have tried to reveal the trends in climate indicators and NDVI based on linear trend regression [1,34]. However, this approach might be insufficient to reveal variations in nonlinear and non-stationary trends, so Extreme-Point Symmetric Mode Decomposition (ESMD) was proposed, which has been proven to be effective in revealing nonlinear trends such as those of climate and vegetation [36,37].

Previous studies have mainly focused on the fluctuation and trends of temperature and precipitation. However, both the frequency or severity of climate extremes have the potential to have widespread impacts on the natural ecology [38]. Particularly, the occurrence of climate extremes might threaten vegetation growth, which has attracted widespread attention [39,40]. Related studies show an intensifying trend in climate extremes in recent decades around the world, including in Europe [41], North America [42], Central Asia [43], East Asia [44,45], and Oceania [46]. Nevertheless, many unknowns remain regarding the correlation between vegetation dynamics and climate extremes. Thus, as for arid and semi-arid regions, the response of vegetation dynamics to climate change should give priority to climate extremes. 

As the core area of the Silk Road Economic Belt, Xinjiang occupies a vital strategic position in China’s economic development. However, the vegetation could be very sensitive to climate change in such a weak ecological environment, which has attracted widespread attention from scientists, governments, and the public [24]. Therefore, the goals of this study were to: (1) monitor the spatiotemporal change of NDVI and Land-use/Land-cover (LULC); (2) analyze the effects on the NDVI and LULC by the climate change; and (3) reveal the response of NDVI to climate extremes.

## 2. Material and Methods

### 2.1. Study Area

**Figure 1:**
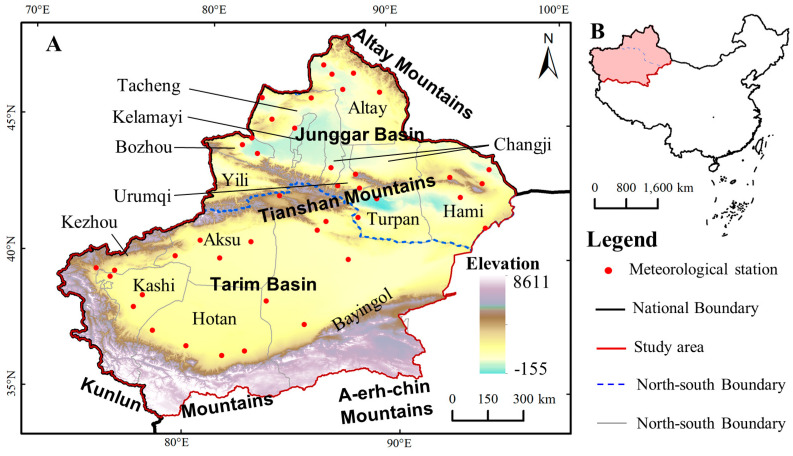
Location of the study area of Xinjiang (**A**) in China (**B**). The figure was mapping based on the Standard Map NO.GS (2016)2923, which could be downloaded from the Ministry of Natural Resources in China (http://bzdt.ch.mnr.gov.cn/). The elevation data was collected from Resource and Environment Data Cloud Platform of China (http://www.resdc.cn/AchievementList1.aspx).

The study site was the Xinjiang Uygur Autonomous Region of China (Xinjiang, for short), located on China’s northwest at 73.40°–96.18° E and 34.25°–48.10° N, see in Figure 1. The total land area is 1.66 million km^2^, accounting for almost one-sixth of China’s land area. Owing to its deep inland location on the border of Central Asia, Xinjiang is a typical arid region, with a long-term average annual precipitation of 150 mm, only about 25% of the average level in China. There are two main basins lying between three high mountains, with the order from north to south being Altay Mountains, Junggar Basin, Tianshan Mountains, Tarim Basin, Kunlun and A-erh-chin Mountains [47]. These high mountains could block the entrance of water vapor into the large basins [31]. Tianshan mountain lies in the central part of Xinjiang and divides it into North Xinjiang (NXC, for short) and South Xinjiang (SXC, for short) [39].

The vegetation is mainly distributed in the mountains and oases [17], while there is significant spatial heterogeneity between NXC (Grassland and Desert Vegetation dominant) and South Xinjiang (Alpine and Desert Vegetation dominant). Most vegetation stops growing in winter. So, we chose May to September as the vegetation growing season, and divided it into Spring (May), Summer (from June–August), and Autumn (September).

### 2.2. Data Collection and Processing

**Figure 2:**
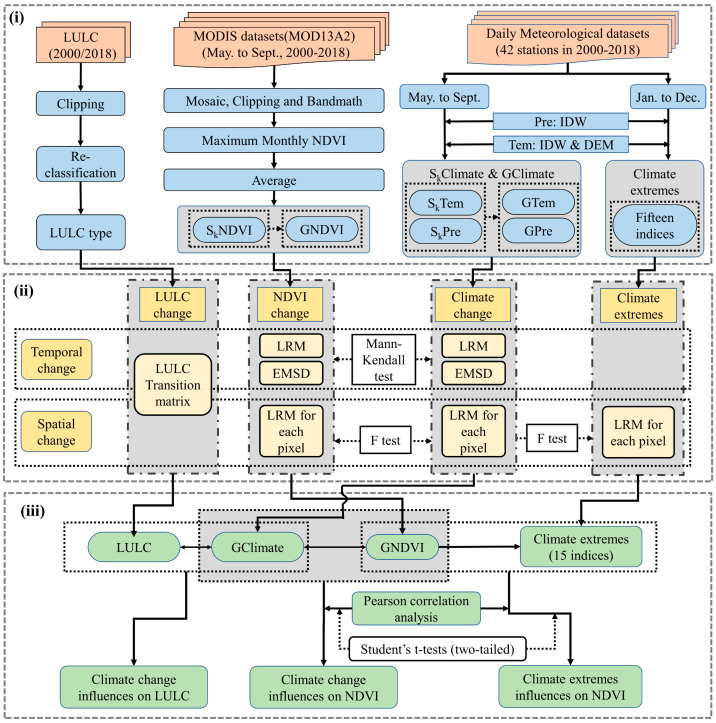
Technical flow chart. (**i**) Data preprocessing, (**ii**) spatio-temporal characteristics of the NDVI and climate change, and (**iii**) the effects on the NDVI and LULC by the climate change. Tem and Pre denotes the temperature and precipitation, respectively. S_k_ includes S_1_, S_2_, S_3_, and S_4_, represents spring, summer, autumn, and growing seasons, respectively. IDW is the Inverse Distance Weighting; ESMD is the Extreme-point Symmetric Mode Decomposition; LRM is the Linear Regression Method.

The data processing roadmap is shown in Figure 2i.

#### 2.2.1. MODIS Time-Series Datasets

The MODIS-NDVI-16 day-1 km product (MOD13A2, with a spatial resolution of 1 km and a temporal resolution of 16 days) could be downloaded in the National Aeronautics and Space Administration (NASA, https://search.earthdata.nasa.gov). In total 1140 remote sensing images with orbit numbers H23V04, H23V05, H24V04, H24V05, H25V04, and H25V05 and covering the period from 2000–2018 were downloaded. Then the data were spliced and the coordinate system was registered to the World Geodetic System 1984 (WGS 84) in batches by the MODIS Reprojection Tool (MRT for short, NASA, Washington, DC, USA). The images were clipped using the boundary vector file of the study area. Furthermore, the NDVI value unit of the original data was 10^−4^, so they still needed to be multiplied by 10^−4^.

The Monthly Maximum NDVI (M*_j_*NDVI *_i_*) could be obtained by the tools of Maximum Value Composite in ENVI 5.3. The equation is shown in formula (1):

(1) $$M_{j}{\mathrm{NDVI}}_{i}=\max({\mathrm{NDVI}}_{ij1},{\mathrm{NDVI}}_{ij2})$$

where NDVI*_ij_*_1_ and NDVI*_ij_*_2_ represent the NDVI in the first and second halves of month *j* in year *i*, respectively; M *_j_*NDVI*_i_* denotes the maximum NDVI of month *j* in year *i*. The *i* represents 1 for the year 2000, 2 for the year 2001, and so on, while *j* is 5 for May, 6 for June, and so on.

Furthermore, M*_j_*NDVI *_i_* was averaged to obtain the average seasonal NDVI (S_k_NDVI_i_, k = 1,2,3 represents spring, summer, and autumn respectively) and the average NDVI in the growing season (GNDVI*_i_*). The *i* indicates the year from 2000–2018. Previous studies regarded areas of NDVI being less than 0.1 as non-vegetation covered areas (NVCA) [17,39], which could be extracted by Mask using ArcGIS 10.6 (Environmental Systems Research Institute, Redlands, CA, USA). 

The continuously observed daily meteorological data in 2000–2018 were obtained from the National Meteorological Data Center of China (http://data.cma.cn/). The climate indices in the year *i* could be calculated based on daily data, including the yearly average temperature in spring (S_1_Tem*_i_*), Summer (S_2_Tem*_i_*), Autumn (S_3_Tem*_i_*) and the Growing Season (GTem*_i_*); and the yearly precipitation in Spring (S_1_Pre*_i_*), Summer (S_2_Pre*_i_*), Autumn (S_3_Pre*_i_*) and the Growing Season (GPre*_i_*). Then the climate indices from 42 meteorological stations (MS) were interpolated into planar raster by Inverse Distance Weighting (IDW). Notably, temperature could be affected by latitude, longitude, and altitude. Therefore, the interpolation of temperature should be combined the IDW modify with Digital Elevation Model (DEM) [48] by Equation (2):

(2) $$\left\{ \begin{array}{l} T_{h}=T_{0}+A\times H\\ T_{dem}=T_{s}-A\times H_{dem} \end{array}\right.$$

where *T_h_* is the temperature modified to DEM = 0; *T*_0_ and *H* are the temperature and DEM of MS. *A* is temperature drop rate (= 0.491 °C/100 m) [48]. *T_s_* is the results of IDW of *T_h_*. *H_dem_* is the DEM raster data, which could be download from Resource and Environment Data Cloud Platform of China (http://www.resdc.cn/AchievementList1.aspx). *T_dem_* is the results of temperature interpolation by IDW modified with DEM. 

#### 2.2.2. Land-Use/Land-Cover (LULC) Datasets

The LULC Dataset was collected from the Resource and Environment Data Cloud Platform (http://www.resdc.cn/data/), which was released by the Chinese Academy of Sciences. Then, we download the LULC datasets in 2000 and 2018, and extracted the study area by Mask using ArcGIS 10.6. The raster dataset had 25 types, with a spatial resolution of 1 km. For the actual situation in Xinjiang, the LULC types were re-divided into twelve types, including Forest, Shrub, Water, Grassland (high coverage), Grassland (moderated coverage), Grassland (low coverage), Permanent snow, Cultivated land, Construction land, Sandy desert, Gobi desert, and Bare land.

### 2.3. Methods

The technology roadmap is shown in Figure 2ii,iii.

#### 2.3.1. Inter-Annual Change Analysis and Mann–Kendall Test

The Extreme-point Symmetric Mode Decomposition (ESMD) is an adaptive signal decomposition method developed by Hilbert-Huang transformation [37]. The data could be decomposed from high to low frequency to generate a series of intrinsic mode functions together with an adaptive global mean curve. [14]. The ESMD could separate the interannual and general climate trends [36]. The ESMD was implemented with the Java-based ESMD4j v1.8 software (Qingdao University of Technology, Qingdao, PRC). The main steps of the software: (1) Set the sampling interval equal to 1. (2) Select the minimum number of residual mode extremum points and the maximum number (≤40) of iterations, and then calculate the variance ratio to determine the optimal number of filters. (3) Decompose and calculate the mode and generate the adaptive global average (ESMD trend). Besides the ESMD, the Linear Regression Method (LRM) was used to analyze the trends of climate and NDVI [49].

The Mann–Kendall Test is a non-parametric method that is used to detect trends in a time series; it can eliminate outliers and reduce the impact of missing data [13,20]. Therefore, it has been widely used to test long time series trends [50]. 

For the sequence X = (*x*_1_, *x*_2_, …, *x*_n_ ), the magnitude relation of *x_i_* and *x_j_* was first determined for all dual values. Then the null hypothesis denotes the data in the sequence are randomly arranged, with no significant trend. Otherwise, the alternative hypothesis denotes the sequence has a trend of increasing or decreasing. In this study, trends with *p* values less than 0.05 were considered to be significant. The Mann–Kendall statistic S is given by Equation (3):

(3) $$S=\sum_{i=1}^{n-1}\sum_{j=i+1}^{n}sign(x_{j}-x_{i})$$

where n is the number of sequence samples, and *x*_i_ and *x*_j_ are time points *i* and *j*, respectively. *sign*(*x*_j_ − *x*_i_) is a sign function calculated by Equation (4):

(4) $$sign(x_{j}-x_{i})=\left\{ \begin{array}{ll} 1 & (x_{j}-x_{i})>0\\ 0 & (x_{j}-x_{i})=0\\ -1 & (x_{j}-x_{i})<0 \end{array}\right.$$

The results of the Mann–Kendall statistic Z approximately follow a standard normal distribution and can be applied to test the significance of the trend. The Z value is given by Equation (5):

(5) $$Z=\left\{ \begin{array}{ll} (S-1)/\sqrt{Var(S)} & if\;S>0\\ 0 & if\;S=0\\ (S+1)/\sqrt{Var(S)} & if\;S<0 \end{array}\right.$$

where *Var*(*S*) is expressed by Equation (6).

(6) $$Var(S)=\frac{1}{18}\left[n\left(n-1\right)\left(2n+5\right)-\sum_{i=1}^{m}t_{i}(t_{i}-1)(2t_{i}+5)\right]$$

where *m* is the number of tied groups, and *t*_i_ is the number of observations in the *m*th group.

In a bilateral trend test for a given confidence level α, if |Z| < Z_1-α/2_, then the null hypothesis is accepted, which indicates that the variation trend of the time series data is not significant at α. Conversely, the null hypothesis is rejected, which indicates that there is a significant increasing (Z > 0) or decreasing (Z < 0) trend at α.

#### 2.3.2. Spatial Change Analysis

The NDVI’s slope (*θ*_slope_) was the interannual variability of seasonally integrated NDVI over a specific time period using least-squares line fitting [26,51]. The equation is shown in formula (7):

(7) $$\theta_{\mathrm{slope}}=\frac{n\times \sum_{i=1}^{n}\left(i\times {\mathrm{NDVI}}_{iq}\right)-\sum_{i=1}^{n}i\times \sum_{i=1}^{n}{\mathrm{NDVI}}_{iq}}{n\times \sum_{i=1}^{n}i^{2}-{\left(\sum_{i=1}^{n}i\right)}^{2}}$$

where *i* is 1 for the year 2000, 2 for the year 2001, and so on. *n* is the total years (*n* = 19). NDVI*_iq_* is the NDVI of pixel *q* in year *i*, including the GNDVI, and S_k_NDVI. If *θ*_slope_ > 0, this indicates that NDVI increased in 2000–2018. Otherwise, it indicates a decreasing trend.

Similarly, the yearly spatial change of temperature and precipitation could also be calculated with the Equation (8):

(8) $$C_{\mathrm{slope}}=\frac{n\times \sum_{i=1}^{n}\left(i\times C_{iq}\right)-\sum_{i=1}^{n}i\times \sum_{i=1}^{n}C_{iq}}{n\times \sum_{i=1}^{n}i^{2}-{\left(\sum_{i=1}^{n}i\right)}^{2}}$$

where C denotes the interpolation result of temperature or precipitation by Inverse Distance Weighting (IDW), including the GTem, GPre, S_k_Tem, and S_k_Pre. C*_iq_* is the C of pixel *q* in year *i*. If C_slope_ > 0, this indicates that climate indices increased in 2000–2018. Otherwise, it indicates a decreasing trend.

The F test was applied to test the trend’s significance. We referred to the critical value table of F-distribution and calculated an F value equal to 4.38 at the level of α = 0.05. Combined with the results of *θ*_slope_ and F tests, the trend of the NDVI could be divided into four types: improved significantly (*θ*_slope_ > 0, *p* < 0.05), improved but not significantly (*θ*_slope_ > 0, *p* > 0.05), degraded but not significantly(*θ*_slope_ < 0, *p* > 0.05), degraded significantly (*θ*_slope_ < 0, *p* < 0.05).

#### 2.3.3. Climate Extremes

In total, twenty-seven core indices have been defined exactly by the Expert Team on Climate Change Detection and Indices (ETCCDI) (http://etccdi.pacificclimate.org/) [20,44]. These indices could reflect the different aspects of extreme climate in different regions [43,52]. As not all indices were meaningful, some indices were eliminated and fifteen extreme climate indices were selected for analysis in this study (see Table 1). The definitions could be seen in the report by the ETCCDI (http://etccdi.pacificclimate.org/definition.shtml). 

The extreme climate indices for each year were calculated by RClimdex 1.0 (Climate Research Branch of Meteorological Service of Canada, Downsview, ON, Canada), an R editor-based software. Moreover, to ensure the credibility of the results, the data were strictly quality controlled by RClimdex 1.0 before calculating.

#### 2.3.4. Pearson Correlation Coefficient

Each pixel was analyzed spatially to obtain the correlation between the GNDVI and climate change, the GNDVI and extreme climate indices, respectively. The Pearson Correlation Coefficient (*r_xy_*) was measured using formula (9):

(9) $$r_{xy}=\frac{\sum_{i=1}^{n}\left(x_{i}-\bar{x})(y_{i}-\bar{y}\right)}{\sqrt{\sum_{i=1}^{n}{\left(x_{i}-\bar{x}\right)}^{2}}\sqrt{\sum_{i=1}^{n}{\left(y_{i}-\bar{y}\right)}^{2}}}$$

where *x_i_* and *y_i_* are the NDVI and climate index values in the growing season of the year *i*, respectively. $\bar{x}$ and $\bar{y}$ are the long-time average annual value of the GNDVI and climate index values, respectively. *n* is the time series length. The *r* values range from (−1)–1, and the larger the absolute value of *r* is, the stronger the correlation.

Then, student’s *t*-tests (two-tailed) could be applied using SPSS Statistics 22 (IBM Corp., Armonk, NY, USA) [5], so as to detect whether *r_xy_* was significant or not. 

## 3. Results

### 3.1. Spatiotemporal Distribution of Climate

#### 3.1.1. Temperature 

**Figure 3:**
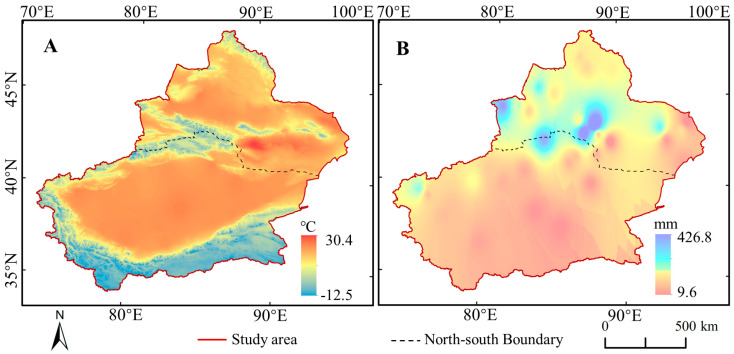
Yearly average temperature (**A**) and precipitation (**B**) for growing season in Xinjiang during 2000–2018. The Figure was mapping based on the daily meteorological data in 2000–2018 from the National Meteorological Data Center of China (http://data.cma.cn/).

Figure 3A shows the average temperature in the growing season (GTem) of 2000–2018 in Xinjiang. Owing to the large altitude span, the distribution shows the characteristics of low GTem in mountains and high Gtem in basins, ranging from (−12.5 °C)–30.4 °C. Thereinto, the GTem were below 10 °C in most areas of Tianshan, Kunlun and A-erh-chin Mountains. The area of GTem exceeding 20 °C in Tarim Basin and Junggar Basin reached 89.26% and 64.25%, respectively. 

**Figure 4:**
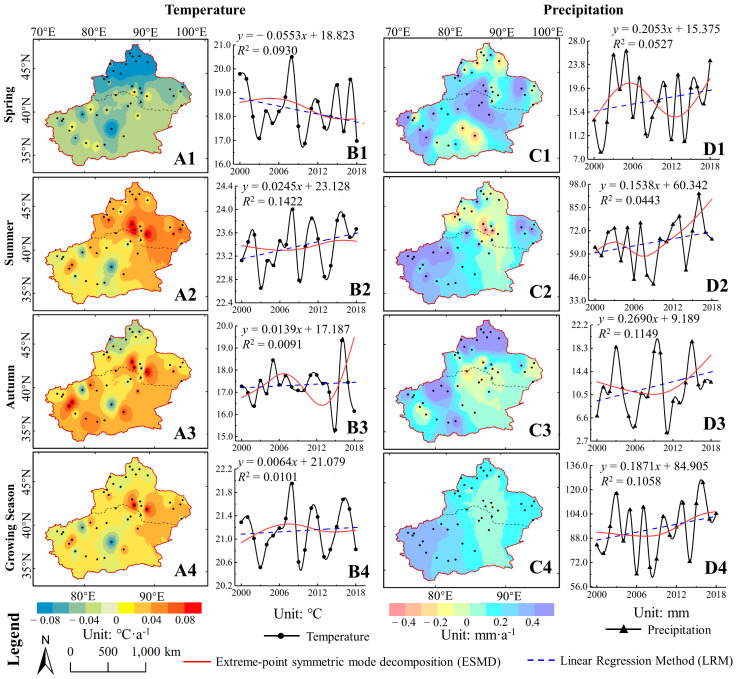
Spatial-temporal change for temperature and precipitation in Xinjiang during 2000–2018. (**A**,**C**) denote the spatial pattern for the change of temperature and precipitation, respectively. (**B**,**D**) are the interannual variations and the trends of temperature and precipitation, respectively. 1~4 denotes the in spring, summer, autumn, and the growing season, respectively.

The variation trends of temperature had different spatial pattern in different seasons, as presented in Figure 4A1–A4. For growing seasons, a high proportion of the area where the GTem has increased, accounted for about 70.93% of the total area of Xinjiang. The GTem of the Altai Mountains, the central Tarim Basin, and the western Kunlun Mountains showed decreasing trends, while the Junggar Basin, the Tianshan mountains, and the A-erh-chin Mountains showed increasing trends. Among these, Urumqi, Turpan, and Kashi had obvious increases in GTem, with growth rates of 0.11, 0.10, and 0.06 °C·a^−1^, respectively. In spring, the average temperature in spring (S_1_Tem) has decreased, with higher decreasing trend in NXC but lower in SXC. In summer, the increasing trend of average temperature in summer (S_2_Tem) in NXC was stronger than that in SXC. And the distribution of the average temperature in autumn (S_3_Tem) trend was approximately consistent with that of the GTem.

Figure 4B1–B4 display the temporal variation of temperature in 2000–2018. Both the ESMD and LRM showed increasing trends in the GTem. Furthermore, the ESMD observed that the GTem variation fluctuates, with a trend of first increasing (in 2000–2007 with a rate of 0.047 °C·a^−1^) and then decreasing (in 2008–2014, with a rate of −0.024 °C·a^−1^) and then increasing (in 2015–2018, with a rate of 0.012 °C·a^−1^). As for different seasons, the S_1_Tem showed a significant decline trend with a rate of 0.0553 °C·a^−1^ (*p* < 0.1), whereas the S_2_Tem and the S_3_Tem increased by 0.0245 °C·a^−1^ (*p* < 0.1) and 0.0139 °C·a^−1^, respectively.

#### 3.1.2. Precipitation

The average precipitation in the growing season (GPre) of North Xinjiang (NXC) was higher than that of South Xinjiang (SXC), as illustrated in Figure 3B. In SXC, the proportion of area with GPre ≤ 100 mm accounted for about 87.65%, while the proportion of area with GPre ≤ 50 mm accounted for about 39.62%. By contrast, the GPre in NXC was mostly in the range of 100–150 mm. Moreover, the GPre around Urumqi and Tianchi reached the highest (426.86 mm).

Figure 4C depicts the change of the precipitation spatial pattern in 2000–2018. The GPre in Kezhou, Aksu, and Kashi increased significantly, reaching 0.6685, 0.6315, and 0.6173 mm·a^−1^, respectively. The GPre of Urumqi in NXC showed a decreasing trend, with a rate of 0.3251 mm·a^−1^.

However, the spatial variation of precipitation are different in different seasons.

(a) The S_1_Pre in the vicinity of the Tianshan Mountains tended to increase, while other distribution characteristics were similar to those in the growing season.
(b) The increasing trend of S_2_Pre in Xinjiang resembled that of GPre.
(c) The area with an increasing trend in S_3_Pre in Xinjiang, NXC, and SXC was roughly the same, with proportions of 81.62%, 82.93%, and 80.48%, respectively. Unlike other seasons, there was an obviously high value for the increasing trend of S_3_Pre around the Altai Mountains (1.476 mm·a^−1^).

The interannual variation in precipitation for Xinjiang in 2000–2018 is shown in Figure 4D. The GPre showed an increasing trend with a linear rate of 0.1871 mm·a^−1^. Moreover, the ESMD curve showed that the trend of GPre was smooth with a slight decrease from 2000–2007 and an increase from 2008–2018 (with a rate of 0.3483 mm·a^−1^). As for different seasons, the average precipitation for each year in Spring (S_1_Pre), Summer (S_2_Pre), and Autumn(S_3_Pre) indicated an increasing trend, among which the S_3_Pre increased by 0.2690 mm·a^−1^ (*p* < 0.1). The trend rates of S_1_Pre and S_2_Pre were 0.2053 and 0.1538 mm·a^−1^, respectively.

#### 3.1.3. Climate Extremes

**Figure 5:**
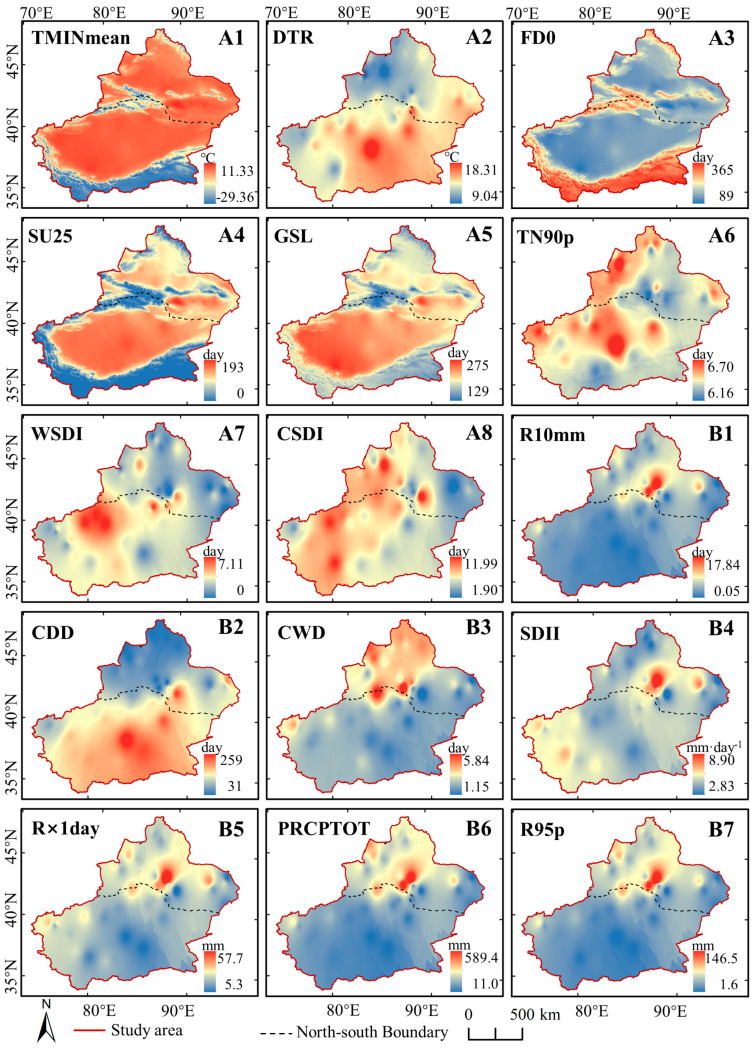
Yearly average extreme climate indices in Xinjiang during 2000–2018. The meanings of the abbreviation are as follows: Mean Minimum Temperature (TMINmean); Diurnal temperature range (DTR); Frost day (FD0); Summer days (SU25); Growing season length (GSL); Warm nights (TN90p); Warm speel duration index (WSDI); Cold speel duration index (CSDI); Number of heavy precipitation days (R10mm); Consecutive dry days (CDD); Consecutive wet days (CWD); Simple daily intensity index (SDII); Maximum 1-day precipitation (R × 1day); Wet day precipitation (PRCPTOT); Very wet day precipitation (R95p).

Figure 5 shows the average value of the 15 extreme climate indices during 2000–2018. The A1–A8 were temperature extremes. Thereinto, the distribution of TMINmean, SU25, and GSL was consistent with that of GTem, while the FD0 was opposite to that of GTem. The TMINmean has great spatial differentiation in Xinjiang, with the highest value of 11.33 °C and lowest value of −29.36 °C. Besides the high-altitude mountainous areas, the FD0 was mostly 120 d–180 d, with the frost period generally from November–March of the next year. The SU25 of NXC was mostly 60 d–120 d, and only the Turpan and Hami were more than 150 d. However, the SU25 varied greatly in SXC, ranging from 0 d–193 d with greater values in the Tarim Basin and lower values in the high-altitude mountainous areas. The DTR showed a large temperature difference between day and night in Xinjiang ranging from 9.04–18.31 °C, with the DTR of SXC was higher than that of NXC. There was no obvious difference (<1 d) for TN90p. Furthermore, it showed an east-west difference for the distribution of both the WSDI and the CSDI.

As for precipitation extremes (see B1–B7 in Figure 5), there was little difference between SDII (6.07 mm·d^−1^) and CWD (4.69 d), indicating the overall drought in the study area. The results of CDD indicated that SXC was drier than NXC, with higher than 100 d in most area of SXC and lower than 100 d in most area of NXC. Furthermore, the distribution of R10mm, R × 1day, PRCPTOT, and R95 was consistent with that of GPre. The results show that SXC had not only the lower precipitation but also the lower rainfall intensity than NXC. The areas with high rainfall intensity were mainly located around Urumqi.

Table 2 illustrates the variation in extreme indices of temperature was stronger than that of precipitation. The Mann–Kendall test showed that the number of frost days (FD0) significantly decreased (*p* < 0.05), the simple daily intensity index (SDII) significantly increased (*p* < 0.05), and the number of warm nights (TN90p) significantly increased (*p* < 0.1). These three indices—FD0, SDII, and TN90p—varied at rates of −4.221 d·(10a)^−1^, 0.315 mm·(d·10a)^−1^, and 0.744 d·(10a)^−1^, respectively. Furthermore, the trends in the extreme precipitation indices of wet day precipitation (PRCPTOT) and very wet day precipitation (R95p) also displayed quick but insignificant rates of increase, with rates of 13.909 mm·(10a)^−1^ and 7.318 mm·(10a)^−1^, respectively. 

### 3.2. Spatiotemporal Distribution of NDVI

**Figure 6:**
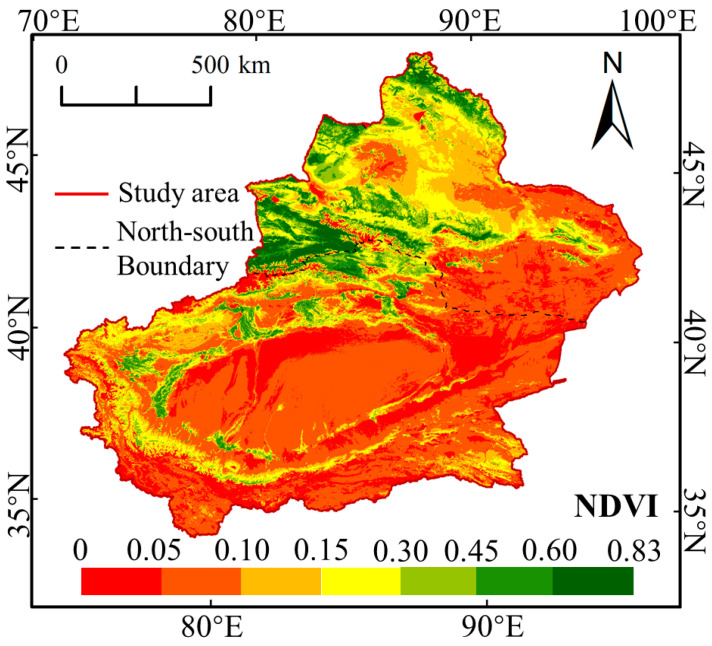
Yearly average NDVI for growing season in Xinjiang during 2000–2018.

Figure 6 reveal evident variations in average NDVI in the Growing Season (GNDVI) in Xinjiang during 2000–2018, ranging from 0–0.83 with greater values in the north and lower values in the southwest. Areas with high vegetation coverage generally exhibited an NDVI of over 0.6. However, these areas only covered 2.61% of the total study area, which primarily found in Altay and Tianshan Mountains, such as Yili, Bozhou, Altay, and Tarbagatay. Furthermore, the NDVI of less than 0.1 could be regarded as the non-vegetation covered areas (NVCA), which accounted for 58.01% of the total area, mainly located in Tarim Basin and east of Junggar Basin.

**Figure 7:**
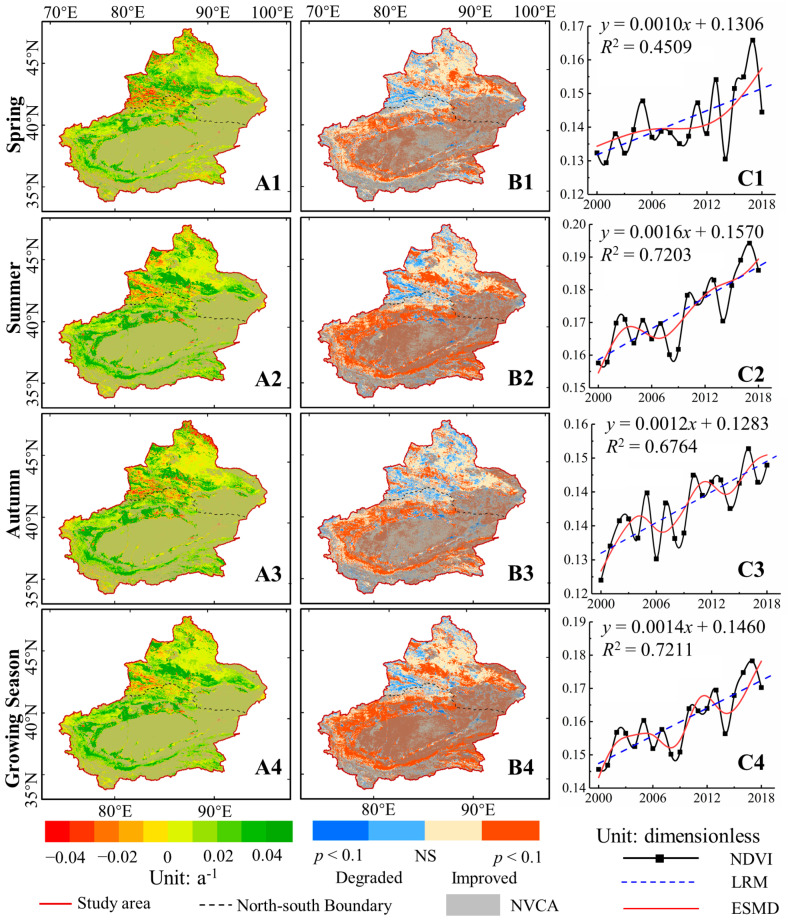
Spatial-temporal change of NDVI in Xinjiang during 2000–2018. (**A**) and (**B**) are the variation and its significance of NDVI. (**C**) denotes the interannual variations and the trends of NDVI. The NS represents the correlation is not significant. The NVCA denotes the non-vegetation covered areas.

Figure 7A,B presents the spatial map of NDVI variation trend and its significance (*p* < 0.05), respectively. The part of the study area except NVCA was vegetation coverage area (VCA). Overall, the tendency of NDVI were heterogeneous for the spatial patterns, but homogeneous for different seasons. In NXC, the areas with significantly improved GNDVI were mainly distributed in the northern margin of Tarim Basin and the southern margin of Junggar Basin, accounting for 29.90% and 33.54% of the VCA. In addition, the proportion of significantly degraded areas of GNDVI accounted for 1.72% of the VCA; these areas were scattered in the northern foothills of the Tianshan Mountains and at the edge of the Altai Mountains. The degraded areas were mainly located in the transition zone between desert and oasis. Owing to the Taklimakan Desert located in the Tarim Basin, the VCA of SXC was low, with area proportions of 29.84% for GNDVI. Even so, there was still a high proportion of improved areas GNDVI (90.63%) in SXC. Among them, significant increases (*p* < 0.05) in GNDVI (60.47%) also accounted for a high proportion of the area, which mainly distributed at the margin of Tarim Basin, especially along the Tarim River.

Figure 7C shows the temporal variation of NDVI in 2000–2018. Both LRM and ESMD displayed an increasing trend of GNDVI, with linear slopes of 0.0014 a^−1^ (*p* < 0.01). Additionally, The ESMD curve depicted an interannual growth trend was observed in all other years except for the slight decreases in 2006–2008 and 2013–2014. For different seasons, the average seasonal NDVI all increased and reached 1% significance level. The ESMD curves exhibited increasing but fluctuant trends in S_1_NDVI, S_2_NDVI, and S_3_NDVI, with the linear rates of 0.001, 0.0016, and 0.0012 a^−1^, respectively.

### 3.3. Spatiotemporal Distribution of LULC

**Figure 8:**
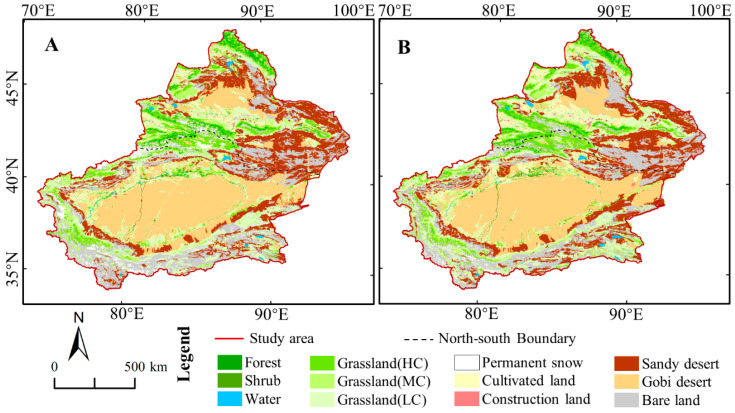
The LULC of Xinjiang in 2000 (**A**) and 2018 (**B**). HC, MC, and LC denote the high coverage, moderated coverage, and low coverage, respectively.

Figure 8 and Table 3 shows the LULC of Xinjiang in 2000 (A) and 2018(B). Xinjiang had the highest proportion of grassland, accounting for about 1/3 of the total area. Among them, the grassland coverage of NXC (about 35%) was higher than that of SXC (about 26%). Low coverage grassland was mainly distributed in SXC. Additionally, the total area of Sandy desert, Gobi desert and Bare land accounted for about 2/3 of the total. Notably, the Sandy desert was mainly distributed in the Tarim Basin of SXC, while the area of Gobi Desert was mainly distributed in the Junggar Basin of NXC.

Table 4 depicted the transfer matrix of LULC during 2000–2018. The area of Cultivated land changed the most in Xinjiang, increasing by 30,834 km^2^ in 2000–2018, which indicated the rapid development of agriculture in Xinjiang. The area of Construction land had doubled, which mainly concentrated in NXC. Notably, the area of Permanent snow has been reduced by 20,436 km^2^ and more than 80% of them were in SXC. The proportion of Permanent snow transferred into Bare land and Grassland accounted for 71.5% and 28.0%, respectively. The Grassland (LC) was both the largest area of transfer-in and transfer-out for all LULC types in Xinjiang and SXC. Furthermore, the area of Grassland (HC) increased by 17,984 km^2^ with three main sources, including the transfer of Grassland (MC) and Grassland (LC), the transfer of Permanent snow and bare land from SXC, the transfer of Forest from NXC. The Sandy Desert and Gobi Desert areas seemed to be stable in Xinjiang, but the equilibrium is an illusion of due to the sharply decrease in SXC and great increase in NXC.

### 3.4. Climate Changes Affects on NDVI and LULC

#### 3.4.1. Climate Change influences on NDVI

The NDVI responded differently to temperature and precipitation for different seasons in Xinjiang, as presented in Table 5. The GNDVI was positively correlated with temperature and precipitation in Xinjiang. However, NDVI had a stronger response to precipitation in the growing season, which indicates that the improvement of vegetation was mainly affected by the increase in precipitation. Furthermore, the response of NDVI to precipitation was much higher than that of temperature in spring and summer. The correlation between S_1_NDVI and temperature was very low in spring. This means the increase in precipitation in spring and summer was beneficial to the growth of vegetation. For autumn, the correlations between S_3_NDVI and temperature increased (*p* < 0.1). Meanwhile, the response of S_3_NDVI to temperature was slightly higher than that of precipitation.

**Figure 9:**
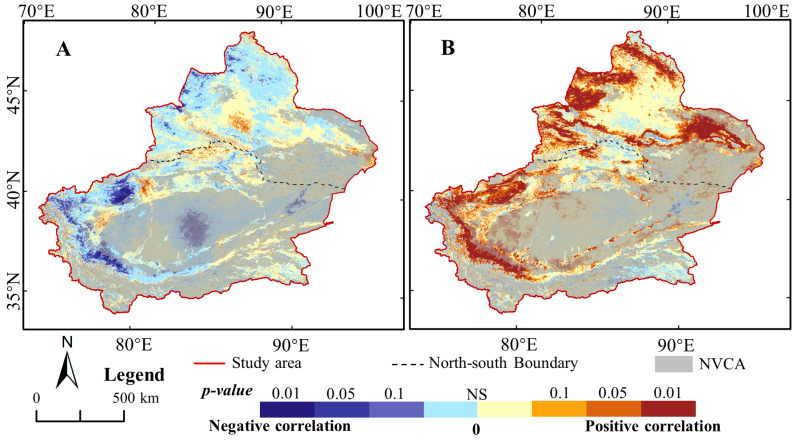
Regional difference of the Pearson correlation coefficients between the GNDVI and climate in growing seasons of Xinjiang. (**A**) and (**B**) denote the temperature and precipitation, respectively. NS denotes the correlation is not significant. The NVCA denotes the non-vegetation covered areas.

Figure 9A illustrates that the GNDVI was insignificantly correlated to the inter-annual variability of GTem in the majority of the study area, with the average coefficient of 0.082. The correlation coefficients between GNDVI and GTem were negative in the majority of VCA and were significant negative, especially in the VCA of SXC. Conversely, the regions with significant positive correlation were mainly located in Tianshan, Hami and Aksu.

In the majority of the VCA, the GNDVI had significant and positive correlations to precipitation, as presented in Figure 9B, but it had weak and negatively correlations to temperature, indicating that the precipitation affected strongly on GNDVI than temperature during the past 19 years. Concretely, the significant and positive correlations of GNDVI and precipitation was observed greatly in the mountains and basins, such as in the Aksu, Hotan, Kashi, Hami, Bozhou, and Altay. Therefore, the restrain of rising temperature on vegetation will weaken the promoting of increasing precipitation, with the warming and wetting evolvement trend of the climate for Xinjiang in the future.

#### 3.4.2. Climate Change influences on LULC

With the climate warming, the Permanent snow have melted in large area, resulting in an increase of 14,628 km^2^ of Bare land, 77.5% of which was located in SXC. Furthermore, the area of grassland increased by 5720 km^2^ due to the water nourishment of melting ice and snow. However, these areas were mainly located in the mountainous region of high altitude, such as Tianshan and Kunlun Mountains. Due to the increase of precipitation, the desert ecosystems such as sandy land, Gobi and bare land have also been improved, with a total of 12,863 km^2^ transferred into grassland. Moreover, the desert ecosystem seems to have become more suitable for cultivation, with 10,452 km^2^ converted into cultivated land.

#### 3.4.3. Climate Extremes Influences on NDVI

**Figure 10:**
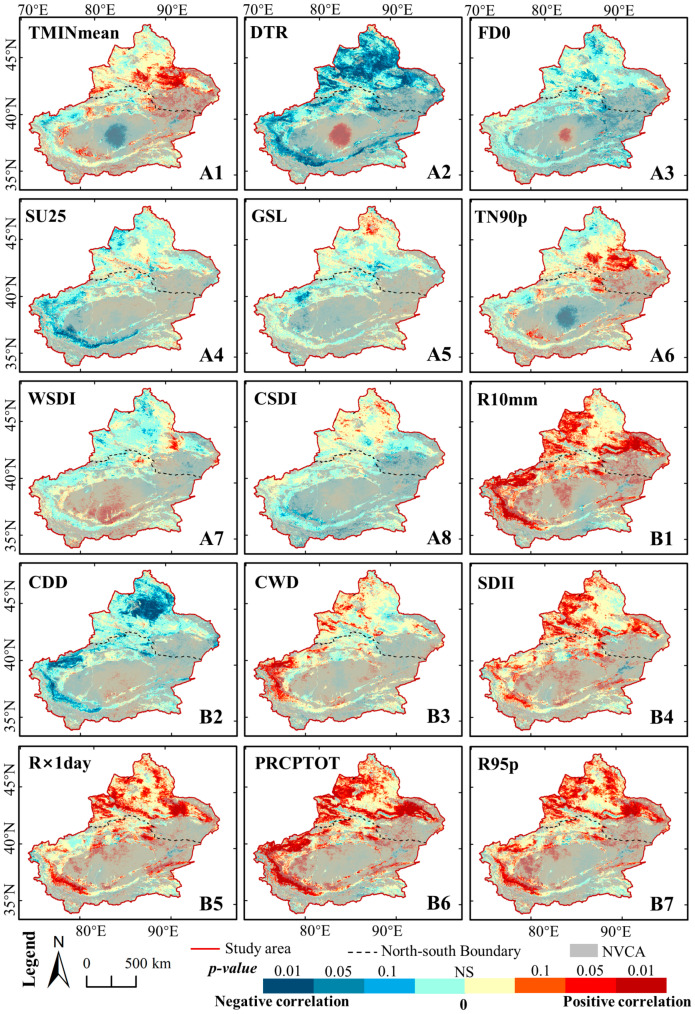
Pearson correlation coefficients between GNDVI and extreme climate indices in Xinjiang. The meanings of the indices are same as Figure 5. NS denotes the correlation is not significant. The NVCA denotes the non-vegetation covered areas.

Table 6 and Figure 10 show the correlation between GNDVI and climate extremes. As for the temperature extremes, the DTR was significantly negatively correlated with NDVI (with a correlation coefficient of 0.634), reaching a significance level of 0.01. Spatially, the DTR with higher negative correlations were mainly located in NXC, indicating that an increase in the temperature difference between day and night could have a bad effect on vegetation growth in NXC. Moreover, the TMINmean was significantly positively correlated with NDVI, reaching a significance level of 0.1 with coefficients of 0.429. These results indicate that night temperature is critical to vegetation growth in Xinjiang. The correlations between the NDVI and the other five extreme temperature indices were insignificant. Notably, the FD0 and TN90p showed negative correlations with NDVI, where the coefficient was equal to −0.341 and 0.311, respectively. It could support the view that the decrease in the frost period favored the growth of vegetation. The SU25 with higher negative correlations were mainly located in the southern margin of Tarim Basin, illustrating that the longer time of summer could inhibit the vegetation growth especially in SXC.

The GNDVI had a stronger correlation to extreme indices of precipitation than that of temperature. Six extreme precipitation indices were significantly positively correlated with NDVI, of which five indices (SDII, R10mm, R × 1day, R95p, and PRCPTOT) reached a significance level of 0.01. Among these, the indices SDII was the most closely correlated with the NDVI, with correlation coefficients of 0.772. Furthermore, the correlation coefficients between the NDVI and the indices of R × 1day, R10mm, R95p, and PRCPTOT were 0.758, 0.751, 0.721, and 0.689, respectively. Spatially, these areas were mainly located in the Tianshan mountain, the southern margin of Tarim Basin, and the western and eastern margin of Junggar Basin. 

These values suggest that the concentrated rainfall could be conducive to vegetation growth in Xinjiang. One index (CWD) reached a significance level of 0.1 with a correlation coefficient of 0.267. This indicates that a continuous humid environment is more suitable for vegetation growth in Xinjiang.

## 4. Discussion

### 4.1. Response of NDVI and LULC to Climate Change

The variation in climate extremes was enhanced over the past 19 years, with the characteristics of more concentrated precipitation and higher temperatures at night. These results confirm that the climate gradually developed a warmer and more humid pattern in Xinjiang, which confirms previous claims [24,39,53,54,55]. Similar studies have reported a significant wetting tendency in northern Xinjiang [4,56], which is consistent with the trend observed in this study. However, unlike some studies revealed a trend of dryness in Xinjiang from 2000–2015 [32]. This might be caused by differences in the selection of research indicators and scales. Additionally, vegetation growth has improved significantly in the past 19 years in Xinjiang. The observation of a vegetation increase in recent decades is consistent with the results of the dynamic greening trend for vegetation in Eurasia, Central Asia, and Western China in previous studies [27,28,56,57]. These variations have led to the optimistic expectation that the fragile eco-environment in arid regions can be improved [24].

Spatially, both temperature and precipitation have tended to increase over the past 19 years, with the variation being higher in NXC than in SXC, which is consistent with previous studies [36,55]. Furthermore, the temperature increase in summer was particularly noticeable in NXC, especially in Urumqi, Turpan, and Hami. These urban areas might act as heat islands, exacerbating the warming trend [53]. The precipitation trend in autumn tended to be an increase in the Altay Mountains and a decline in Junggar Basin, which is consistent with previous research [24,36]. As a previous study reported [11,55], the regions of vegetation showing obvious restoration were mainly distributed in the Tianshan Mountains, Altay Mountains, and around the margins of Tarim Basin. A similar result was also found in this paper. The vegetation degradation area was mainly located at the intersection of desert and oasis, which might have been caused by the lack of water supply. A previous study found that the NDVI decreased significantly in Taklimakan Desert of Tarim Basin [55].

The spatial patterns of the NDVI were positively affected by both temperature and precipitation change. Spatially, NXC is more sensitive to precipitation than SXC. The little precipitation and strong evaporation rate in Xinjiang could have large effects on vegetation growth [24]. Furthermore, in dry conditions, vegetation might reduce the carbon supply to bacterial communities which, in turn, limits the growth of vegetation [58,59,60]. These results confirm previous findings concerning the drought risk in arid regions, which revealed that precipitation is the primary climatic driver for vegetation changes [2,25,33].

The rising temperatures might enhance the vegetation growth of Xinjiang by two aspects. Firstly, a properly increasing temperature might extend the growing season of vegetation. For example, the response of the NDVI to temperature was slightly higher than that of precipitation in autumn. This result agrees with previous work [12,35] and provides further evidence that precipitation and temperature have different effects on vegetation growth in different seasons. Secondly, an increasing temperature would accelerate the glacial ablation of the high mountains [54,55], and then the runoff might promote the growth of vegetation. The results of the transfer matrix of LULC could vindicate this judgment.

The response of NDVI to the extreme index of precipitation was stronger than that of temperature. Meanwhile, the response of the NDVI to the climate extremes was stronger than the response to climate change. Extreme drought might be more likely to decrease vegetation growth and even ecosystem productivity and stability [8]. Furthermore, the NDVI variation in arid regions was eventually determined by the precipitation increase, especially by precipitation extremes [39]. Therefore, extreme precipitation could be regarded as a vital factor in the variation of NDVI in Xinjiang, especially for continuous concentrated precipitation. As for temperature extremes, the results indicated that the mean minimum temperature (TMINmean) and warm nights (TN90p) had significant positive correlations with the NDVI, which is consistent with previous research [39,43,44,52]. Furthermore, we did find that the diurnal temperature range (DTR) was significantly negatively correlated with the NDVI, especially in NXC. This illustrates that the significant enhancement of vegetation was consistent with the significant increase in the night temperature in Xinjiang. Thus, higher night-time temperatures in the study area could regulate and enhance vegetation growth by reducing the frost risk and increasing vegetation respiration.

### 4.2. Suggestions, Limitaion, and Prospects

The vegetation in arid areas could be more sensitive to climate change, which might influence the eco-environment in the countries and regions of the Belt and Road [4]. The work could enrich the understanding of the effects of climate change on land cover change and vegetation dynamics, laying the basis for its sustainable management. Consequently, the advantages and disadvantages of climate change and its influence on vegetation should be fully comprehended by the local government.

(a) As for the advantages, climate change might create an environment that is more suitable for specific types of vegetation. For example, the grassland showed the highest levels of improvement, with these areas showing positive responses to an increase in precipitation. These findings could support a scientific basis for the implementation and management of ecological restoration programs to improve the fragile environment. The government could use the advantages of vegetation growth from climate change to implement some ecological restoration strategies (e.g., enhancing the protection of grassland especially during periods of increased precipitation).
(b) Regarding the disadvantages, an increase in temperature will accelerate the melting of glaciers on high mountains, which could nurture and enhance the vegetation growth. However, it could also exacerbate water shortages and increase the Bare land, which would threaten the fragile local arid ecosystems. Thus, the local government should carry out effective measures to tackle climate warming, such as increasing energy conservation and emission reduction efforts. Notably, Xinjiang is the National Large-scale Coal Mining Base of China, where the carbon emissions of coal consumption cannot be ignored. Therefore, the local government should actively optimize the structure of energy utilization.

Because of the complexity of the vegetation and its response to climate change, there were still some limitations in this study. Owing to the topographic relief, and the sparse MS which mainly in or around cities, which could affect the accuracy of the results. Notably, the irrational anthropogenic socioeconomic activity could disturb the growth of vegetation, such as the increment of Cultivated land occupied the grassland of 19,483 km^2^. Thus, further research should be done to quantitatively analyze the coupling mechanism between climate change, vegetation growth, and human activities.

## 5. Conclusions

This work proves and evidences the effect of climate change on land cover change and vegetation dynamics, laying the basis for its sustainable management. Since climate change showed a warming trend, the Permanent snow has been reduced by 20,436 km^2^. The NDVI exhibited an increased volatility trend, with the significant improvement regions mainly located in the margin of basins. The humid trend could provide more suitable conditions for vegetation growth and ecological restoration, but the warmer might prevent vegetation growth. The response of NDVI to precipitation was stronger than the response to temperature. Spatially, NXC was more sensitive to precipitation than SXC. Seasonally, the response of NDVI to precipitation was higher than the temperature in spring and summer; but in autumn, it was the opposite. Continuous concentrated precipitation could be considered as a vital factor for vegetation dynamics in Xinjiang. Furthermore, the significant enhancement of vegetation was consistent with the significant increase in night-time temperature. Therefore, the reduction in the diurnal temperature range and higher night-time temperatures could enhance vegetation dynamics.

## Figures and Tables

**Table 1:** Definitions of extreme climate indices used in this study.

Temperature			Precipitation		
Abbreviation	Index Name	Unit	Abbreviation	Index Name	Unit
TMINmean	Mean Minimum Temperature	°C	R10mm	Number of heavy precipitation days	d
DTR	Diurnal temperature range	°C	CDD	Consecutive dry days	d
FD0	Frost days	d	CWD	Consecutive wet days	d
SU25	Summer days	d	SDII	Simple daily intensity index	mm·d^−1^
GSL	Growing season length	d	R × 1day	Maximum precipitation per day	mm
TN90p	Warm nights	d	PRCPTOT	Wet day precipitation	mm
WSDI	Warm speel duration index	d	R95p	Very wet day precipitation	mm
CSDI	Cold speel duration index	d			

**Table 2:** The variation trends of the extreme climate indices.

Table			Precipitation		
Index	Rate	Unit	Index	Rate	Unit
TMINmean	0.294	°C·(10a)^−1^	R10mm	0.592	d·(10a)^−1^
DTR	−0.211	°C·(10a)^−1^	CDD	0.718	d·(10a)^−1^
FD0	−4.221 **	d·(10a)^−1^	CWD	0.013	d·(10a)^−1^
SU25	−0.578	d·(10a)^−1^	SDII	0.315 **	mm·(d·10a)^−1^
GSL	2.335	d·(10a)^−1^	R × 1day	2.254	mm·(10a)^−1^
TN90p	0.744 *	d·(10a)^−1^	PRCPTOT	13.909	mm·(10a)^−1^
WSDI	−0.205	d·(10a)^−1^	R95p	7.318	mm·(10a)^−1^
CSDI	−0.891	d·(10a)^−1^			

Note: significant at *—p < 0.1, and **—*p* < 0.05, respectively. The meanings of the abbreviations are the same as in Figure 5.

**Table 3:** Statistics of the LULC in 2000 and 2018 (km^2^).

Area	ID	1	2	3	4	5	6	7	8	9	10	11	12
Xinjiang	2000	59,419	20,941	17,260	114,276	116,699	246,208	9269	38,235	4295	404,838	294,385	309,218
	2018	90,253	13,721	13,908	132,260	110,666	239,079	11,357	17,799	8621	405,168	294,932	297,279
	Change	+30,834	−7220	−3352	+17,984	−6033	−7129	+2088	−20,436	+4326	+330	+547	−11,939
NXC	2000	32,477	17,328	6812	65,756	50,903	79,386	3474	5954	2858	58,502	168,582	104,617
	2018	47,628	10,758	3880	75,667	47,614	87,372	4382	2215	5663	50,985	147,471	113,014
	Change	+15,151	−6570	−2932	+9911	−3289	+7986	+908	−3739	+2805	−7517	−21,111	+8397
SXC	2000	26,930	3603	10,441	48,501	65,775	16,6794	5795	32,217	1437	346,335	125,753	204,813
	2018	42,619	2961	10,022	56,550	63,036	15,1661	6975	15,556	2958	354,184	147,422	184,450
	Change	+15,689	−642	−419	+8049	−2739	−15133	+1180	−16,661	+1521	+7849	+21,669	−20,363

Note: The ID means: 1, Cultivated land; 2, Forest; 3, Shrub; 4, Grassland (HC); 5, Grassland (MC); 6, Grassland (LC); 7, Water; 8, Permanent snow; 9, Construction land; 10, Sandy desert; 11, Gobi desert; 12, Bare land.

**Table 4:** Transfer matrix of LULC in 2000 and 2018 (km^2^).

Xinjiang	Year	2000												Transfer in
Year	ID	1	2	3	4	5	6	7	8	9	10	11	12	
2018	1	48,122	677	2567	3764	7983	13,073	525		1928	6171	4323	1120	42,131
	2	46	6160	797	4672	1183	376	136	32	2	136	70	111	7561
	3	1262	1450	2564	1958	2201	2530	122	3	69	1347	221	181	11,344
	4	899	9930	3307	69,926	22,949	7944	479	3557	71	1641	1027	10,530	62,334
	5	1766	1178	2809	18210	37,463	26,150	413	1147	83	4786	2539	14,122	73,203
	6	2672	574	2448	6677	28,962	102,422	493	1765	188	13,273	23,438	56,167	136,657
	7	320	201	184	420	675	808	5991	9	13	1479	806	451	5366
	8		4	1	72	350	327		13180			5	3860	4619
	9	3170	41	146	134	244	1050	80		1657	555	1276	268	6964
	10	300	130	1625	998	3719	34,455	455		78	35,7077	4034	2297	48,091
	11	652	93	587	476	2531	26,291	465	54	165	13,822	222,393	27,403	72,539
	12	210	503	225	6969	8439	30,782	110	18,488	41	4551	34,253	192,708	104,571
Transfer out		11,297	14,781	14,696	44,350	79,236	143,786	3278	25,055	2638	47,761	71,992	116,510	
**NXC**	**Year**	**2000**												**Transfer in**
**Year**	**ID**	**1**	**2**	**3**	**4**	**5**	**6**	**7**	**8**	**9**	**10**	**11**	**12**	
2018	1	26,023	309	859	1847	3530	7201	180		1097	3162	2597	823	21,605
	2	36	5567	399	3853	612	87	79	2	2	33	32	56	5191
	3	126	975	448	1078	480	443	29	1	9	50	120	121	3432
	4	586	8657	2273	44,048	11,890	2817	189	427	49	522	449	3760	31,619
	5	1273	818	1360	8113	20,770	10,347	129	62	66	833	1355	2488	26,844
	6	1972	313	907	2518	10,861	39,154	157	74	144	3760	13,961	13,551	48,218
	7	186	141	49	190	159	203	2491	3	10	534	266	150	1891
	8		4		41	19	23		1469				659	746
	9	1843	29	99	96	168	877	15		1250	190	854	242	4413
	10	117	36	145	133	288	7213	120		69	41,209	1072	583	9776
	11	217	41	150	101	297	5629	39		130	5873	124,909	10,085	22,562
	12	98	438	123	3738	1829	5392	46	3916	32	2336	22,967	72,099	40,915
Transfer out		6454	11,761	6364	21,708	30,133	40,232	983	4485	1608	17,293	43,673	32,518	
**SXC**	**Year**	**2000**												**Transfer in**
**Year**	**ID**	**1**	**2**	**3**	**4**	**5**	**6**	**7**	**8**	**9**	**10**	**11**	**12**	
2018	1	22,094	368	1708	1917	4453	5871	345		831	3009	1726	297	20,525
	2	10	592	397	819	571	289	57	30		103	38	55	2369
	3	1134	475	2115	879	1720	2086	93	2	60	1297	101	60	7907
	4	310	1265	1029	25,868	11,050	5125	290	3130	22	1118	578	6765	30,682
	5	492	360	1449	10,093	16,688	15,803	284	1085	17	3953	1184	11,628	46,348
	6	699	260	1541	4157	18,095	63,254	336	1690	44	9513	9477	42,595	88,407
	7	134	60	135	230	516	605	3500	6	3	945	540	301	3475
	8			1	31	331	302		11,688			5	3198	3868
	9	1327	12	47	38	76	173	65		407	365	422	26	2551
	10	183	94	1480	865	3431	27,242	335		9	315,869	2962	1714	38,315
	11	435	52	437	375	2234	20,661	426	54	35	7948	97,452	17,313	49,970
	12	112	65	102	3229	6610	25,383	64	14,532	9	2215	11,268	120,861	63,589
Transfer out		4836	3011	8326	22,633	49,087	103,540	2295	20,529	1030	30,466	28,301	83,952	

Note: The meaning of the ID is same as Table 3.

**Table 5:** Pearson correlation coefficient between the NDVI and climate for different seasons in Xinjiang from 2000–2018.

Index	Temperature	Precipitation
S_1_NDVI	−0.082	0.538 ***
S_2_NDVI	0.276 *	0.747 ***
S_3_NDVI	0.321 *	0.278 *
GNDVI	0.082	0.797 ***

Note: significant at *—*p* < 0.1, **—*p* < 0.05, and ***—*p* < 0.01. The S_1_NDVI, S_2_NDVI, S_3_NDVI, and GNDVI denotes the NDVI in spring, summer, autumn, and growing season.

**Table 6:** Statistics of the correlations between the GNDVI and extreme climate indices in the vegetation coverage area (VCA) of Xinjiang.

Type	Extreme Indices	Percentage (%)								Pearson Correlation Coefficient
		NC ***	NC **	NC *	NC	PC	PC *	PC **	PC ***	
Temperature	TMINmean	0.10	0.61	1.01	26.67	54.89	6.25	6.85	3.62	0.429 *
	DTR	15.45	19.87	10.98	39.95	12.44	0.43	0.72	0.17	−0.634 ***
	FD0	1.02	3.57	4.08	59.80	29.82	0.87	0.68	0.16	−0.341
	SU25	1.28	4.13	4.23	52.18	35.22	1.45	1.17	0.33	−0.082
	GSL	0.34	1.52	1.75	45.86	47.04	1.83	1.40	0.26	0.133
	TN90p	0.11	0.57	0.97	32.70	51.80	5.23	5.76	2.85	0.311
	WSDI	0.16	1.22	2.16	52.96	40.16	1.76	1.35	0.23	−0.037
	CSDI	0.20	1.19	1.77	44.98	48.45	2.02	1.23	0.16	−0.187
Precipitation	R10mm	0.09	0.40	0.50	11.72	46.43	9.70	15.06	16.10	0.751 ***
	CDD	7.25	10.11	7.11	53.44	21.20	0.50	0.33	0.06	−0.317
	CWD	0.12	0.50	0.71	25.03	57.86	6.09	6.48	3.21	0.286
	SDII	0.08	0.32	0.41	13.26	56.78	10.12	12.74	6.28	0.771 ***
	R × 1day	0.12	0.39	0.44	13.37	51.50	10.77	14.08	9.33	0.758 ***
	PRCPTOT	0.12	0.41	0.46	10.32	42.05	9.97	15.71	20.96	0.689 ***
	R95p	0.06	0.31	0.40	12.64	48.10	11.47	16.07	10.95	0.721 ***

Note: significant at *—*p* < 0.1, **—*p* < 0.05, and ***—*p* < 0.01, respectively. PC and NC represent the positive and negative correlations, respectively. The meanings of the abbreviations are the same as in Figure 7.
